# Editorial: The role of immunotherapy in melanomas

**DOI:** 10.3389/fonc.2023.1293040

**Published:** 2023-09-28

**Authors:** Anna Fateeva, Suzie Chen

**Affiliations:** ^1^ Susan Lehman Cullman Laboratory for Cancer Research, Rutgers University, Piscataway, NJ, United States; ^2^ Graduate Program in Cellular and Molecular Pharmacology, Rutgers University, Piscataway, NJ, United States; ^3^ Rutgers Cancer Institute of New Jersey, New Brunswick, NJ, United States

**Keywords:** melanoma, immunotherapy, biomarkers, combinatorial treatments, adverse effects, toxicity

Melanoma is the deadliest form of skin cancer that had very limited treatment options until 2011 when targeted inhibitors were approved for melanoma, first against mutated BRAF and subsequently against MEK, which significantly improved treatment outcomes. In 2011, the first immunotherapy regimen, ipilimumab – an antibody against CTLA-4, was approved for melanoma treatment paving the path for the use of immunotherapies in other cancer types. Several years later, immune checkpoint inhibitors (ICI) against PD-1 and PD-L1 were approved. They increased response to the treatment in comparison to other available therapies; however, their efficacy still remained to be improved (1, 2). As such, combinations of different agents were explored and found to enhance response rates and survival. However, combinational therapies almost inevitably cause increased adverse effects (3). Therefore, it remains crucial to develop new improved combinational therapies for melanoma treatment and explore biomarkers that could be used to predict treatment efficacy and refine therapeutic regimens for patients in a personalized manner.

Eight articles were selected for this Research Topic highlighting current advances in immunotherapy regimens for the treatment of melanoma. Below, we first discuss the works focusing on the development of new therapeutic regimens; then, we summarize articles dealing with the discovery of new prognostic biomarkers for patients’ response to therapy.

Vázquez-Montero et al. summarized the current knowledge on the combination of nivolumab, an anti-PD-1, and ipilimumab, an anti-CTLA-4, for melanoma treatment. In their review, the authors highlighted clinical trial results and the combination efficacy, as well as suggested patient groups that might benefit more from the combination rather than a single-therapy regimen.

Silk et al. reported a phase 1b clinical trial of a combinational treatment consisting of pembrolizumab, an anti-PD-1 agent, and IL-2 in patients with unresectable or metastatic melanoma. The goal of the trial was to establish the maximum tolerated dose (MTD) of IL-2 when co-administered with pembrolizumab. As a result of the study, MTD was not reached rendering high dose IL-2 therapy in combination with pembrolizumab tolerable. However, the study was conducted in a small number of participants leaving room for further evaluations to make definitive conclusions.

Ma et al. discussed a clinical case of multiple primary malignant neoplasms, one of them – acral melanoma. There are no established treatment guidelines for this rare form of melanoma and the presence of multiple primary tumors; the patient was treated with a combination of chemotherapy and immune checkpoint inhibitors. A missense mutation in exon 2 of the KRAS gene was discovered, and the authors hypothesized that previous adjuvant chemotherapy could be the cause of melanoma formation. This report showcases the need for continued efforts to develop options for patients with multiple primary tumors.

A novel combination was explored by Tsarovsky et al. using an intratumoral injection of hu14.18-IL2 immunocytokine (IC) – an anti-GD2 antibody linked to interleukin 2 –, local radiotherapy (RT) and cyclophosphamide (CY), a chemotherapeutic agent, to produce an enhanced T-cell-mediated antitumor response. CY was shown to increase the efficacy of the combination of IC and RT by decreasing the levels of regulatory T-cells (Tregs) and upregulating the ratio of CD8^+^ cells/Tregs as assessed by flow cytometry. Interestingly, the authors described an immunological memory, which manifested in the mice rejecting the tumor when rechallenged 60 days after the primary tumor rejection.

Another combinatorial treatment was analyzed by Stoff et al. – an anti-PD-1 together with lenvatinib, a multi-targeted tyrosine kinase inhibitor. This combination seems promising based on the results of its efficacy not only in cutaneous melanoma, but also rare forms of melanoma – acral, mucosal and uveal; patients with more aggressive disease progression and metastases also benefitted from the regimen. However, the authors noted toxic side effects in a significant number of patients.

As highlighted in some of these studies, toxic side effects are one of the consequences of combination of immunotherapy with other treatment regiments in melanoma. Being able to identify patients who would benefit the most from immunotherapy treatment would be highly valuable in choosing optimal therapeutic regime depending on the risk the patients are facing, which would reduce undesirable side effects in some patients and decrease the economic burden.

UV exposure from the sun is a known environmental contributor in skin cancer. Liu et al. investigated a correlation between sun exposure and effectiveness of immunotherapy in patients with metastatic melanoma. The authors stratified patients into groups based on the tumor location to represent differential exposure to sun of various body parts and treatment regime. They analyzed and compared survival differences for patients that underwent treatment in the years before and after the introduction of ICI in the clinic. Increased sun exposure for the patients with head and neck melanomas was observed to positively correlate with the effectiveness of ICI, as opposed by the lack of it in patients with acral melanoma that arises in the sites minimally exposed to the sunlight. Additionally, nomograms were established for predicting 3- and 5-year overall survival for patients.

Another study on the prognostic factors was conducted by Moschella et al. in which the authors analyzed blood cell profiles in patients before and after treatment using a combination of peptide vaccine and low-dose interferon-α2b. The authors used multiparametric flow cytometry to determine the baseline immune profiles in patients; they identified circulating T- and NK-cell subsets with varying presence in pre- and post-treatment samples. Further analyses established correlation of some of these markers with relapse-free survival and overall survival. Three immune cell subsets – regulatory T cells, CD56^dim^CD16^-^ NK cells and central memory γδ T-cells – were distinguished as especially reliable markers as confirmed by principal component analysis and receiver operating characteristic curves.

Similarly, Nurzat et al. were able to single out a prognostic biomarker CCR6 to predict response to immunotherapy using multiple analytical techniques, including single cell RNA sequencing, functional enrichment analysis and Cox regression analysis. CCR6 was established to positively correlate with various immune checkpoint protein levels, immune cell population enrichment and associate with favorable outcomes in patients with cutaneous melanoma.

The articles included in this Research Topic summarized and discussed benefits and shortcomings of existing immunotherapies for melanoma, spotlighted several novel combinatorial immunotherapies and identified potential biomarkers as means of providing patients with more personalized treatment regimens.

## Author contributions

SC: Conceptualization, Funding acquisition, Writing – review & editing. AF: Conceptualization, Writing – original draft, Writing – review & editing.
